# Serum ApoB/ApoA1 ratio in patients with CHB and the occurrence of HBV related cirrhosis and HBV related hepatocellular carcinoma

**DOI:** 10.1038/s41598-024-61820-x

**Published:** 2024-05-14

**Authors:** Xin Cai, Shi Peng, Xuan Xiao, Zhaoyang Huang, Pingan Zhang

**Affiliations:** Department of Clinical Laboratory, Institute of Translational Medicine, Renmin Hospital of Wuhan University, Wuhan, 430060 Hubei People’s Republic of China

**Keywords:** ApoB/ApoA1 ratio, Chronic hepatitis B, Cirrhosis, Hepatocellular carcinoma, Tumour biomarkers, Biomarkers

## Abstract

Clinical research has suggested that chronic HBV infection exerts a certain effect on the occurrence of cardiovascular disease by regulating cholesterol metabolism in liver cells. High serum apolipoprotein B/apolipoprotein A1 (ApoB/ApoA1) ratio plays a certain role in the above regulation, and it serves as a risk factor for cardiovascular disease. However, whether the ApoB/ApoA1 ratio is correlated with chronic HBV infection and its disease progression remains unclear. In accordance with the inclusion and exclusion criteria, all 378 participants administrated at Renmin Hospital of Wuhan University from March 2021 to March 2022, fell into Healthy Control (HC) group (50 participants), Hepatocellular carcinoma (HCC) group (107 patients), liver cirrhosis (LC) group (64 patients), chronic hepatitis B (CHB) group (62 patients), chronic hepatitis C (CHC) group (46 patients) and Hepatitis E Virus (HEV) group (49 patients). Serum ApoA1 and ApoB concentrations were measured at admission, and the ApoB/ApoA1 ratio was determined. The levels of laboratory parameters in the respective group were compared and ApoB/ApoA1 ratios in HCC patients and LC patients with different severity were further analyzed. ROC curves were plotted to analyze the early diagnostic ability of ApoB/ApoA1 ratio for HBV-associated HCC. Logistic regression and restricted cubic spline analysis were used to explore the correlation between ApoB/ApoA1 ratio and LC and HCC risk. A comparison was drawn in terms of ApoB/ApoA1 ratio between the groups, and the result was expressed in descending sequence: HEV group > CHB group > LC group > HCC group > CHC group > HC group, early-stage HCC < middle-stage HCC < advanced-stage HCC, Class A LC < Class B LC < Class C LC. Serum ApoB/ApoA1 ratio combined diagnosis with AFP exhibited the capability of increasing the detection efficacy and specificity of AFP for HCC and AFP-negative HCC. The incidence of LC and HCC in the respective logistic regression model showed a negative correlation with the serum ApoB/ApoA1 ratio in CHB patients (*P* < 0.05). After all confounding factors covered in this study were regulated, the result of the restricted cubic spline analysis suggested that in a certain range, serum ApoB/ApoA1 ratio showed an inverse correlation with the prevalence of LC or HCC in CHB patients. Serum ApoB/ApoA1 ratio in CHB patients may be conducive to identifying high-risk patients for HCC or LC, such that LC and HCC can be early diagnosed and treated.

## Introduction

Hepatitis refers to a prevalent liver disease arising from chronic viral infections. It comprises autoimmune hepatitis, both of which induce ongoing liver damage. There are over 350 million chronic HBV carriers, nearly 71 million people infected with HCV and 20 million people infected with HEV worldwide^1^. Persistent damage to the liver may trigger liver cirrhosis (LC), i.e., a stage of partial loss of liver structure and function and potentially life-threatening complications^2^. Hepatocellular carcinoma (HCC) has been confirmed as the sixth most common cancer worldwide, as well as the third largest cause of cancer death. Chronic inflammation plays an important role in the development of HCC^3^. HBV remain one of the critical global risk factors for HCC, LC, and the progression of LC to HCC thus far^4–6^.

When HBV infected patients develop liver injury, individual gene expression is regulated. Notably, the pathway of lipid biosynthetic enzymes is changing, and blood lipid levels correspondingly lose balance^7^. Existing research has suggested that patients with chronic hepatitis B (CHB) are subjected to lower triglyceride (TG), total cholesterol (TCh), and low-density lipoprotein cholesterol (LDL-C) levels, as well as higher high-density lipoprotein cholesterol (HDL-C) levels compared with healthy controls^8,9^. HBV infection is an independent factor correlated with reduced risk of fatty liver and metabolic syndrome^10^. Abnormal lipid metabolism refers to a risk factor for cirrhosis and liver cancer in patients in the progression of CHB^11–13^. Huang’s research has shown that ApoA-I can interact with the HBV regulatory protein HBx, and this interaction impairs the lipid-binding and aggregation properties of ApoA-I, resulting in cellular cholesterol accumulation that promotes HBV virion release. Notably, overexpression of ApoA-I was found to significantly inhibit HBV secretion, suggesting a potential strategy to suppress HBV infection by stimulating ApoA-I^14,15^.

Apolipoprotein B (ApoB) has been reported as the major structural component of atherogenic LDL particles. It takes on critical significance in binding of LDL particles to the LDL receptor for absorption and LDL-C. Excessive ApoB-containing particles are the main trigger in the atherogenic process^16^. Apolipoprotein A1 (ApoA1) serves as the major protein component of HDL particles, interacting with HDL receptors and stimulates lecithin-cholesterol acyltransferase, thus leading to cholesterol esterification, facilitating reverse cholesterol transport, and preventing atherosclerosis^17^. As revealed by existing research, the higher the ApoB/ApoA1 ratio, the more cholesterol will be deposited on the arterial wall, such that atherogenesis can be triggered, and cardiovascular disease risk will be elevated^16^. In terms of CHB patients, there are evidences of a relatively high risk of platelet activation and atherothrombosis^18,19^. HBV can modulate ApoA1 function through HBx, leading to ApoA1 dysfunction, such as decreased self-association ability, increased carbonyl levels and impaired lipid binding ability^20,21^. HBV suppresses ApoB production by inhibiting microsomal triglyceride transfer protein expression^22^. In brief, the variation of the ApoB/ApoA1 ratio arising from the progress of CHB disease is worth studying, and it can indirectly suggest the effect exerted by the progress of CHB disease on atherosclerosis, and add novel insights for the early diagnosis of LC and HCC. Nevertheless, the correlation between ApoB/ApoA1 ratio and CHB disease progression has been rarely reported. Accordingly, this study placed a focus on the correlation between serum ApoB/ApoA1 ratio and CHB disease progression.

## Patients and methods

### Study population

**Figure 1:**
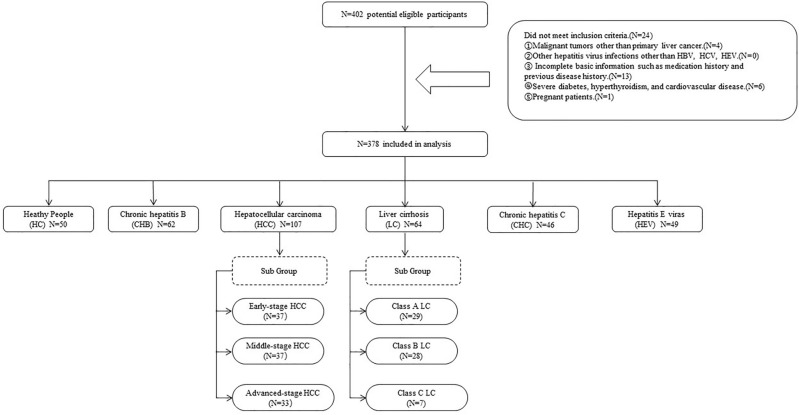
Schematic diagram of grouping of patients.

All 378 participants were recruited from March 2021 to March 2022 in the Department of Infectious Diseases, Department of Intensive Care Medicine, Department of Hepatobiliary Surgery and Department of Oncology at Renmin Hospital of Wuhan University, Wuhan, China. All 378 participants fell into Healthy Control (HC) group (50 participants), HCC group (107 patients), LC group (64 patients), CHB group (62 patients), Chronic hepatitis C (CHC) group (46 patients) and Hepatitis E Virus (HEV) group (49 patients). All patients were confirmed by liver pathological examination, X-ray examination or MRI examination, related virological tests and definite clinical diagnosis were obtained. The inclusion criteria conformed to the guideline of prevention and treatment for chronic hepatitis B: a 2019 update^23^, Chinese guidelines on the management of liver cirrhosis^24^, Chinese guideline for the prevention and control of hepatitis C virus healthcare-associated infection (2022 edition)^25^, standard for diagnosis and treatment of primary liver cancer (2022 edition)^26^, deliberation on the diagnostic criteria for hepatitis E^27^. HCC and LC patients covered were correlated with CHB only, never received any therapy for HCC or LC, and negative in hepatitis B e antigen (HBeAg). All HCC patients had LC. Exclusion criteria for included patients are elucidated as follows: (1) Malignant tumors other than primary liver cancer. (2) Other hepatitis virus infections other than HBV, HCV, HEV. (3) Incomplete basic information such as medication history and previous disease history. (4) Severe diabetes, hyperthyroidism, and cardiovascular disease. (5) Pregnant patients. Figure 1 presents the specific research object inclusion and exclusion details. Figure 1 illustrates the specific research object inclusion and exclusion details.

This study was reviewed and approved by the Medical Ethics Review Committee of Renmin Hospital of Wuhan University (WDRY2022-K117) and approved to exempt patients from informed consent.

### Assessment of liver cirrhosis severity

In all LC patients the severity of illness was assessed using a modified Child–Pugh’s classification. As depicted in Table 1.

**Table 1:** Modified Child–Pugh’s classification.

Clinic and laboratory data	Points for increasing abnormality^1^		
	1	2	3
HE (grade^2^)	None	1–2	3–4
Ascites	None	Mild	Moderate or above
Bilirubin (μmol/L)	< 34.2	34.2–51.3	> 51.3
Albumin (g/L)	≥ 35	28–34	< 28
Prolonged PT (s)^3^	1–4	4–6	> 6

^1^Scoring system: 5–6 points, grade A; 7–9 points, grade B; 10–15 points, grade C. ^2^HE: hepatic encephalopathy; Grade 0: sub-clinical hepatic encephalopathy, SHE, unraveled by Retain tests A or B; Grade 1: anxiety, irritability, depression, impaired concentration, sleep disturbances; Grade 2: disorientation, poor short-term memory, disinhibited behavior, drowsiness; Grade 3: somnolence, bizarre behavior, confusion, amnesia, paranoia; Grade 4: Coma. ^3^PT, Prothrombin Time, also expressed in seconds prolonged < 4; 4–6; > 6.

### Blood sampling and analysis

Three milliliters of fasting venous blood was collected from all study subjects at admission and centrifuged at 3500 r/min (centrifugation radius = 16 cm) for 5 min to separate serum at − 80 °C until use. The levels of alanine aminotransferase (ALT), aspartate aminotransferase (AST), alkaline phosphatase (ALP), γ-glutamyl transpeptidase (GGT), fibronectin (Fn), cholinesterase (CHE), free fatty acid (FFA), urea, TCh, TG, HDL-C, LDL-C, small dense low-density lipoprotein cholesterol (sdLDL-C), lactate dehydrogenase (LDH), and lipoprotein a[LP(a)] were analyzed by enzymatic method using the fully automatic biochemical analyzer ADVIA 2400 (Siemens, Germany). The levels of ApoA-1 and ApoB were determined through a polyethylene glycol (PEG)-enhanced immunoturbidimetric assay. The concentration of alpha fetoprotein (AFP) was detected through the Siemens ADVIA Centaur CP (Siemens).

### Statistical analysis

The experimental data were analyzed with SPSS version 20.0 (IBM Corp, NY), MedCal 15.2.2 (Ostend, Belgium) and GraphPad Prism 6.0 (GraphPad Software, Inc, La Jolla, CA). In accordance with the measurement data, whether the data of the respective group conform to normality was examined using the single-sample Kolmogorov–Smirnov method, the normal distribution data was expressed as χ ± s, the comparison between multiple groups were performed through analysis of variance, and the further pairwise comparison was drawn through LSD-*t* test. The non-normal distribution data were expressed as M (*P*_25_–*P*_75_). The comparison between multiple groups was drawn through the Kruskal–Wallis *H* test, the Mann–Whitney *U* test was performed to draw the comparison between the two groups, and the pairwise comparison was conducted based on the Bonferroni adjustment test level method. The diagnostic value was calculated using the stepwise logistic regression model. *P* < 0.05 indicated a difference that achieved statistical significance. Simple and multiple logistic regression was used to investigate the correlation between ApoB/ApoA1 ratio and LC prevalence in CHB and HCC prevalence in LC. Restricted cubic spline analysis was used to investigate the non-linear correlation between ApoB/ApoA1 ratio and LC prevalence in CHB and HCC prevalence in LC.

### Ethical approval and consent to participate

This study was approved by the Medical Ethics Review Committee of Renmin Hospital of Wuhan University, China, and complies with the Declaration of Helsinki and all participants signed informed consent.

## Results

### Characteristics of study population

Table 2 showed the characteristics of 378 hepatitis participants and the variability of each indicator between groups as superscripts. Among the 233 CHB participants, 64 participants developed LC (27.47%), and 107 participants developed comorbid LC and HCC (45.92%). The Hepatitis E group had the highest levels of AST, ALT, GGT, ALP, Fn, and most of the variations in expression compared with the other groups were statistically significant (*P* < 0.05).

**Table 2:** Characteristics of the study population.

Characteristics	HCC (n = 107)	LC (n = 64)	CHB (n = 62)	CHC (n = 46)	HEV (n = 49)	HC(n = 50)	*H/F*	*P*
Male (%)	57.01	57.81	66.13	47.83	77.55^abd^	42.00^e^	16.60	0.005
Age (years)	57.00 (50.00, 64.00)	53.00 (47.25, 58.75)^a^	48.00 (35.75, 60.25)^a^	58.00 (51.00, 67.00)^bc^	58.00 (49.50, 69.00)^bc^	50.00 (44.25, 54.00)^ade^	37.44	< 0.001
Smoking (%)	34.58	26.56	64.52^ab^	34.78^c^	32.65^c^	0.00^abcde^	53.44	< 0.001
Alcohol drinking (%)	25.23	12.50	62.90^ab^	30.43^c^	28.57^c^	0.00^abcde^	66.22	< 0.001
Chronic hepatitis durations (years)	19.00 (13.00, 23.00)	12.00 (9.00, 17.75)^a^	7.00 (4.00, 12.25)^ab^	2.00 (1.00, 3.00)^abc^	0.00 (0.00, 0.00)^abcd^	0.00 (0.00, 0.00)^abcd^	311.83	< 0.001
Anti-hepatitis (%)	79.44	87.50	88.71	89.13	0.00^abcd^	0.00^abcd^	227.13	< 0.001
AST (U/L)	38.00 (27.00, 75.00)	42.00 (29.00, 64.00)	43.00 (25.00, 92.25)	34.00 (25.00, 52.25)	65.00 (31.50, 180.00)^abcd^	20.00 (16.25, 21.75)^abcde^	94.87	< 0.001
ALT (U/L)	30.00 (25.00, 43.00)	37.00 (22.00, 63.00)	33.00 (19.75, 78.25)	29.50 (16.50 ~ 56.25)	78.00 (30.50 ~ 235.50)^abcd^	18.00 (13.25, 23.75)^abcde^	64.83	< 0.001
ALP (U/L)	118.90 (84.00 ~ 197.20)	94.90 (67.00, 134.70)^a^	100.10 (73.13, 172.00)	129.10 (97.68, 208.53)^bc^	197.58 (120.95, 251.00)^abcd^	59.00 (50.25, 75.75)^abcde^	104.72	< 0.001
GGT (U/L)	98.00 (42.00, 198.00)	52.00 (29.00, 105.00)^a^	80.00 (36.75, 174.25)^b^	66.00 (43.50, 129.00)^a^	132.00 (91.00, 195.00)^abcd^	17.00 (13.00, 23.00)^abcde^	117.92	< 0.001
Fn (mg/L)	217.00 (192.00, 240.03)	201.34 (172.00, 223.96)^a^	219.09 (182.93, 245.91)^b^	217.25 (182.3, 256.35)^b^	251.00 (225.30, 283.68)^abcd^	231.60 (215.55, 248.60)^b^	42.77	< 0.001
CHE (U/L)	6618.00 (4855.00, 8615.00)	4777.00 (3300.00, 7495.00)^a^	6996.50 (4882.00, 9171.50)^b^	6071.00 (3201.75, 8392.75)	6756.00 (3933.50, 8590.00) ^b^	9377.50 (8551.00, 10164.25)^abcde^	59.67	< 0.001
FFA (mmol/L)	0.51 (0.34, 0.83)	0.60 (0.27, 0.97)	0.51 (0.33, 0.74)	0.45 (0.23, 0.64)^ab^	0.44 (0.22, 0.68)^b^	0.26 (0.21, 0.40)^abc^	35.46	< 0.001
Urea (mmol/L)	5.10 (4.36, 6.87)	6.07 (4.93, 7.52)^a^	4.89 (3.31, 6.17)^ab^	5.10 (3.95, 7.25)^b^	5.38 (4.11, 7.18)^c^	5.08 (4.39, 6.12)	17.34	< 0.001
TCh (mmol/L)	4.01 (3.45, 4.67)	3.40 (2.93, 4.29)^a^	4.07 (3.49, 4.66)^b^	3.56 (2.83, 4.64)^a^	4.11 (3.24, 5.11)^b^	4.89 (4.51, 5.20)^abcde^	52.81	< 0.001
TG (mmol/L)	0.98 (0.76, 1.34)	0.99 (0.68, 1.34)	1.31 (1.00, 1.86)^ab^	1.03 (0.75, 1.62)^c^	1.51 (1.20 ~ 2.22)^abd^	1.20 (0.90, 1.52)^e^	38.95	< 0.001
HDL-C (mmol/L)	1.05 (0.80, 1.39)	0.95 (0.63, 1.26)	1.00 (0.68, 1.32)	0.95 (0.74, 1.27)	0.90 (0.45, 1.19)^a^	1.33 (1.20, 1.55)^abcde^	47.23	< 0.001
LDL-C (mmol/L)	2.27 (1.81, 2.88)	1.67 (1.17, 2.36)^a^	2.23 (1.75, 2.90)^b^	2.00 (1.47, 2.96)^b^	2.18 (1.70, 3.09)^b^	2.59 (2.29, 3.02)^b^	39.78	< 0.001
sdLDL-C (mmol/L)	0.50 (0.40, 0.71)	0.33 (0.20, 0.52)^a^	0.57 (0.31, 0.89)^b^	0.45 (0.27, 0.64)^a^	0.57 (0.35, 0.91)^b^	0.69 (0.50, 0.91)^b^	27.58	< 0.001
TCh/HDL-C ratio	4.07 (3.04, 5.04)	3.50 (2.82, 5.21)	4.36 (3.11, 5.56)	3.84 (2.98, 4.78)	4.67 (3.41, 9.19)^abd^	3.59 (2.96, 4.41)^abde^	242.29	< 0.001
Lp(a) (mmol/L)	36.00 (14.00, 70.00)	45.6 (11.63, 88.25)	72.10 (28.03, 211.00)^ab^	72.55 (42.20, 164.68)^ab^	80.10 (34.90, 203.60)^ab^	158.70 (67.55, 244.38)^abcde^	246.16	< 0.001
LDH (U/L)	241.00 (217.00, 318.00)	213.00 (180.25, 254.50)^a^	228.50 (190.00, 260.50)^a^	212.50 (185.00, 237.50)^a^	249.00 (193.50, 307.00)^bd^	182.00 (164.00, 196.75)^abc^	259.65	< 0.001
AFP (ng/ml)	30.45 (5.00, 2182.19)	4.20 (1.91, 12.63)^a^	7.35 (4.65, 12.50)^a^	13.45 (6.28, 21.55)^abc^	12.60 (6.65, 19.70)^abc^	2.90 (2.20, 4.15)^abcde^	94.98	< 0.001
MELD score	9.00 (7.00, 11.00)	9.00 (7.00, 11.00)	8.00 (6.00, 10.00)^ab^	9.50 (6.00, 11.00)	9.00 (7.00, 11.00)^c^	–	8.94	0.063
ApoA1 (g/L)	1.15 (0.93, 1.36)	1.06 (0.80, 1.25)	0.98 (0.83, 1.17)^a^	1.11 (0.99, 1.34)^c^	1.10 (0.68, 1.28)	1.44 (1.34, 1.61)^abcde^	82.41	< 0.001
ApoB (g/L)	0.72 (0.58, 0.82)	0.78 (0.67, 1.05)^a^	0.95 (0.84, 1.09)^ab^	0.69 (0.56, 0.90)^bc^	0.82 (0.66, 1.02)^acd^	0.79 (0.72, 0.92)^c^	46.90	< 0.001

^a^*P* < 0.05, compared with HCC; ^b^*P* < 0.05, compared with LC; ^c^*P* < 0.05, compared with CHB; ^d^*P* < 0.05, compared with CHC; ^e^*P* < 0.05, compared with HEV.

AST, aspartate aminotransferase; ALT, alanine aminotransferase; ALP, alkaline phosphatase; GGT, γ-glutamyl transpeptidase; Fn, fibronectin; CHE, cholinesterase; FFA, free fatty acid; Tch, total cholesterol; TG, triglyceride; HDL-C, high-density lipoprotein cholesterol; LDL-C, low-density lipoprotein cholesterol; sdLDL-C, small dense low-density lipoprotein cholesterol; Lp(a), lipoprotein a; LDH, lactate dehydrogenase; AFP, alpha fetoprotein; MELD, model for end-stage liver disease; ApoA1, Apolipoprotein A1; ApoB, Apolipoprotein B.

### ApoB/ApoA1 ratio is different in serum of patients with various liver diseases and in patients with different stages of HCC and LC

**Figure 2:**
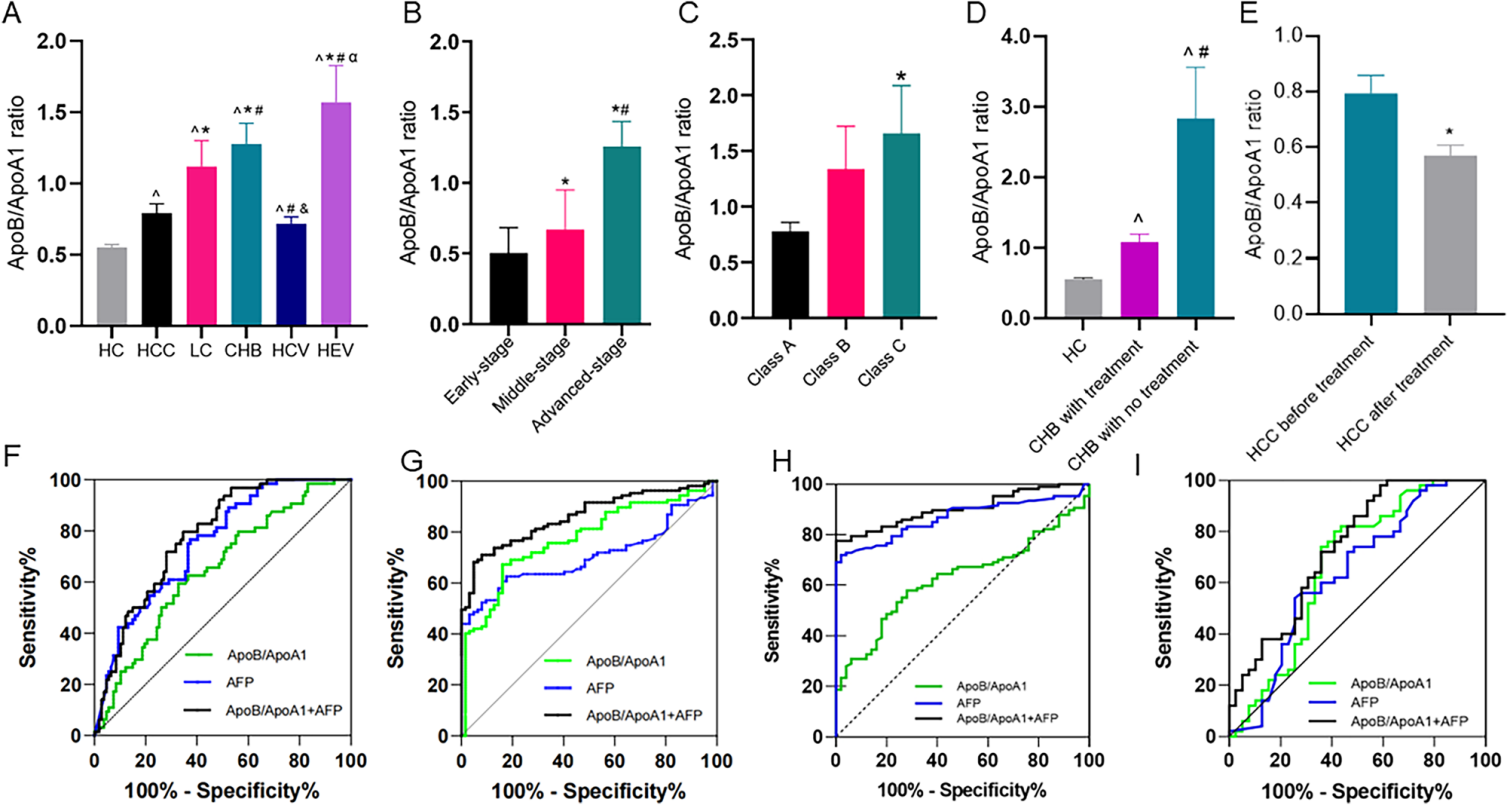
Analysis of ApoB/ApoA1 ratio in different liver diseases and different stages of HCC, LC and HC. (**A**) The ratio of ApoB/ApoA1 in patients with different types of liver diseases and healthy people. ^^^*P* < 0.05, compared with HC group; ^*^P < 0.05, compared with HCC group; ^#^*P* < 0.05, compared with LC group; ^&^*P* < 0.05, compared with CHB group; ^α^*P* < 0.05, compared with CHC group. (**B**) The ratios of serum ApoB/ApoA1 of patients with different stages of HCC. *P < 0.05, compared with early-stage HCC; ^*#*^*P* < 0.05, compared with middle-stage HCC. (**C**) Ratios of serum ApoB/ApoA1 of patients with different Child–Pugh grades of LC. ^*^P < 0.05, compared with Class A liver cirrhosis. (**D**) Ratios of serum ApoB/ApoA1 before and after treatment in patients with CHB.^^^*P* < 0.05, compared with HC group; ^#^*P* < 0.05, compared with CHB with treatment group. (**E**) Ratios of serum ApoB/ApoA1 before and after treatment in patients with HCC. ^*^P < 0.05, compared with HCC group before treatment. (**F**) ROC curve of ApoB/ApoA1 ratio for diagnosis of HCC from LC. (**G**) ROC curve of ApoB/ApoA1 ratio for diagnosis of HCC from CHB. (**H**) ROC curve of ApoB/ApoA1 ratio for diagnosis of HCC from HC. (**I**) ROC curve of ApoB/ApoA1 ratio for diagnosis of AFP-negative HCC from HC.

ApoB/ApoA1 ratio in the serum of individuals with various forms of liver diseases and healthy people varied (Fig. 2A). The ApoB/ApoA1 ratio in the HCC group [0.65 (0.47, 0.85)], which was notably lower than those of the LC group [0.80(0.63, 1.07)] and CHB group [0.93(0.76, 1.30)] (*P* < 0.05). ApoB/ApoA1 ratio reached 0.63(0.51, 0.82), and 0.78(0.59, 1.18) in the CHC group and the HEV group, respectively. The ApoB/ApoA1 ratio of all groups was higher than that of HC group by 0.53 (0.48, 0.64) (*P* < 0.05).

As depicted in Fig. 2B, serum ApoB/ApoA1 ratio in patients suffering from advanced-stage hepatocellular carcinoma [0.89(0.71, 1.26)] were notably higher than those in patients subjected to early-stage hepatocellular carcinoma [0.47(0.35, 0.66)] and those in patients carrying middle-stage hepatocellular carcinoma [0.61(0.49, 0.74)] (*P* < 0.05); serum ApoB/ApoA1 ratio in patients with middle-stage hepatocellular carcinoma were significantly higher than those in patients suffering from early-stage hepatocellular carcinoma (*P* < 0.05). As depicted in Fig. 2C, serum ApoB/ApoA1 ratio in patients with Class C liver cirrhosis [1.40 (0.80, 2.06)] were significantly higher than those in patients with Class A liver cirrhosis [0.69 (0.53, 0.95)] (*P* < 0.05), serum ApoB/ApoA1 ratio in patients with Class B liver cirrhosis [0.82 (0.68, 1.31)] had no statistically significant difference with patients subjected to Class A and Class C liver cirrhosis (*P* > 0.05).

As shown in Fig. 2D and E, serum ApoB/ApoA1 ratio decreased significantly in patients with chronic HBV infection from 2.44 (1.77, 3.40) to 0.92 (0.77, 1.16) after anti-HBV treatment. HCC patients also showed a significant decrease in ApoB/ApoA1 ratio after systemic treatment from 0.71 (0.661, 0.877) to 0.55 (0.45, 0.70).

As depicted in Fig. 2F and G, there is further analysis of the diagnostic role of ApoB/ApoA1 ratio in differentiating HCC from LC or CHB and in combination with AFP, a common diagnostic marker for hepatocellular carcinoma. After combining the ApoB/ApoA1 ratio and AFP, the area under the curve for the combined index was significantly higher compared with the AUC for ApoB/ApoA1 ratio alone (*P* < 0.05). The diagnostic specificity of the combined index was higher than that of ApoB/ApoA1 ratio and that of AFP. As depicted in Tables 3 and 4. As shown in Fig. 2H and Table 5, the ApoB/ApoA1 ratio could reach an AUC of 0.773 (0.703–0.844) and Specificity of 83.87% in the diagnosis of HCC with HC. After combining AFP, the AUC of the combined index was elevated to 0.782 (0.749–0.862), and Specificity was elevated to 95.16%. We analyzed HCC patients with AFP < 10 ng/ml as a separate subgroup in order to further explore the diagnostic value of ApoB/ApoA1 ratio, and tentatively named this group as AFP-negative HCC subgroup. As shown in Fig. 2I and Table 5, the diagnostic AUC of the indicator after the combination of ApoB/ApoA1 ratio and AFP was 0.736 (0.632–0.824), and Specificity could reach 98.00%.

**Table 3:** The performance of ApoB/ApoA1 ratio and AFP for diagnosing HCC from LC.

	AUC (95% CI)	Sensitivity (%)	Specificity (%)	Youden Index	Cut-off
ApoB/ApoA1	0.649 (0.565, 0.733)	59.38	67.29	0.267	0.727
AFP	0.752 (0.714, 0.849)	75.00	63.55	0.386	12.45 ng/mL
Combination	0.861 (0.807, 0.915)	71.88	71.96	0.438	0.550

**Table 4:** The performance of ApoB/ApoA1 ratio and AFP for diagnosing HCC from CHB.

	AUC (95% CI)	Sensitivity (%)	Specificity (%)	Youden index	Cut-off
ApoB/ApoA1	0.773 (0.703–0.844)	67.29	83.87	0.512	0.728
AFP	0.705 (0.629–0.782)	62.62	82.26	0.449	14.20 ng/mL
Combination	0.782 (0.749–0.862)	68.22	95.16	0.634	0.591

**Table 5:** The performance of ApoB/ApoA1 ratio and AFP for diagnosing HCC and AFP negative HCC from HC.

	AUC (95% CI)	Sensitivity (%)	Specificity (%)	Youden index	Cut-off
HCC versus HC					
ApoB/ApoA1	0.773 (0.703–0.844)	67.29	83.87	0.512	0.728
AFP	0.705 (0.629–0.782)	62.62	82.26	0.449	14.20 ng/mL
Combination	0.782 (0.749–0.862)	68.22	95.16	0.634	0.591
AFP negative HCC versus HC					
ApoB/ApoA1	0.670 (0.562–0.766)	58.97	80.00	0.3897	0.67
AFP	0.636 (0.528–0.736)	74.36	54.00	0.2836	3 ng/mL
Combination	0.736 (0.632–0.824)	41.03	98.00	0.3903	0.636

### Logistic analysis of the prevalence of HCC in LC patients and prevalence of LC in CHB patients and ApoB/ApoA1 ratio

To analyze the correlation between ApoB/ApoA1 ratio and HCC in the LC patients in depth, and the correlation between ApoB/ApoA1 ratio and LC in the CHB patients, the data fell into quartiles of ApoB/ApoA1 ratio, with the first quartiles as the reference to determine the odds ratio (OR) for HCC or LC, and the results are listed in Tables 6 and 7. The prevalence of HCC in LC patients and prevalence of LC in CHB patients dispalyed a negative correlation with the ApoB/ApoA1 ratio in the Crude model (*P* < 0.01). After age and gender regulated in Model 1, the results were similar to those of Crude model (*P* < 0.01). The difference continued to achieve statistical significance after further control of HBV infection time, anti-HBV therapy, smoking, drinking, Fn, CHE, FFA, Urea, TCh, TG, HDL-C, LDL-C, sdLDL-C, T/HDL-C, LP(a), LDH, ALT, AST, ALP and GGT. The fully regulated OR in Model 2 was 0.043(95% CI: 0.009, 0.201) for quartile 4 of circulating ApoB/ApoA1 ratio in the LC patients (the highest) versus quartile 1 (the lowest); it reached 0.196 (95% CI: 0.037, 1.033) for quartile 4 of ApoB/ApoA1 ratio in the CHB patients (the highest) versus quartile 1 (the lowest).

**Table 6:** Correlation of prevalence of HCC with serum ApoB/ApoA1 ratios in LC patients.

ApoB/ApoA1 quartile	n	Ratio range	Crude	OR (95% CI)	
				Model 1	Model 2
Quartile 1 (low)	44	≤ 0.5147	Reference	Reference	Reference
Quartile 2	43	0.5147–0.699	0.480 (0.183, 1.259)	0.432 (0.160, 1.165)	0.182 (0.046, 0.710)
Quartile 3	42	0.699–0.8977	0.343 (0.132, 0.890)	0.333 (0.124, 0.893)	0.194 (0.053, 0.717)
Quartile 4 (high)	42	≥ 0.8977	0.234 (0.090, 0.605)	0.205 (0.076, 0.549)	0.043 (0.009, 0.201)
β			− 0.459	− 0.489	− 0.891
SE			0.149	0.153	0.232
*P* for trend			0.002	0.001	< 0.001

Logistic regression was used to examine the correlations between serum levels of ApoB/ApoA1 ratio and HCC in LC patients. Serum ApoB/ApoA1 ratio was divided into quartiles (quartile 4: ≥ 75th, quartile 3: 50–75th, quartile 2: 25–50th, quartile 1: < 25th percentile).

Crude: no adjustment.

Model 1: regulated for age, gender.

Model 2: regulated for the same variables as Model 1 as well as HBV infection time, Anti-HBV therapy, smoking, drinking, Fn, CHE, FFA, Urea, TCh, TG, HDL-C, LDL-C, sdLDL-C, T/HDL-C, LP(a), LDH, ALT, AST, ALP and GGT.

**Table 7:** Correlation of prevalence of LC with serum ApoB/ApoA1 ratios in CHB patients.

ApoB/ApoA1 quartile	n	Ratio range	Crude	OR (95% CI)	
				Model 1	Model 2
Quartile 1 (low)	31	≤ 0.6876	Reference	Reference	Reference
Quartile 2	33	0.6876–0.8626	0.491 (0.174, 1.382)	0.562 (0.196, 1.614)	0.603 (0.122, 2.970)
Quartile 3	30	0.8626–1.1784	0.237 (0.081, 0.693)	0.243 (0.081, 0.727)	0.111 (0.020, 0.604)
Quartile 4 (high)	32	≥ 1.1784	0.280 (0.098, 0.799)	0.278 (0.096,0.806)	0.196 (0.037, 1.033)
β			− 0.446	− 0.461	− 0.637
SE			0.168	0.171	0.264
*P* for trend			0.008	0.007	0.016

Logistic regression was used to examine the correlations between serum levels of ApoB/ApoA1 and LC in CHB patients. Serum ApoB/ApoA1 was divided into quartiles (quartile 4: ≥ 75th, quartile 3: 50–75th, quartile 2: 25–50th, quartile 1: < 25th percentile).

Crude: no adjustment.

Model 1: regulated for age, gender.

Model 2: regulated for the same variables as Model 1 as well as HBV infection time, Anti-HBV therapy, smoking, drinking, Fn, CHE, FFA, Urea, TCh, TG, HDL-C, LDL-C, sdLDL-C, T/HDL-C, LP(a), LDH, ALT, AST, ALP and GGT.

### Restricted cubic spline analysis

Restricted cubic spline analyses found that after adjusting for all confounding factors included in this study, the inflection point occurs when the ApoB/ApoA1 ratio is 0.7, which means that when the ratio of ApoB/ApoA1 was less than 0.70, the prevalence of HCC in LC patients decreased with ratio of ApoB/ApoA1. When the ratio of ApoB/ApoA1 was greater than 0.7, the prevalence of HCC reached a plateau. When the ratio of ApoB/ApoA1 was between 0.51 and 0.86, there was negatively correlation between the prevalence of LC and the ApoB/ApoA1 ratio; when the ratio was greater than 0.86, it was a plateau for the prevalence of LC.

## Discussion

This is the first study to investigate the correlation between ApoB/ApoA1 ratio and the CHB disease progression. Chronic HBV infection refers to a major public health concern. In chronic HBV infection, there may be persistent low-grade liver inflammation with transient high-grade episodes of liver inflammation and activation of fibrotic processes that trigger liver fibrosis and LC, which may eventually lead to decompensated liver disease and HCC in 25—40% of HBV carriers^28^. HBeAg and HBV DNA serve as independent and vital factors for the development of disease complications. In patients subjected to HBV infection in early childhood, most of the sequelae of cirrhosis and hepatocellular carcinoma occur after HBeAg seroconversion^29^. Some existing research has suggested that a direct proportion exists between HBV DNA levels and the risk of LC and HCC development, and the incidence of LC and HCC is tenfold lower in people with persistently controlled low levels of HBV DNA than in those with high levels of HBV DNA^30,31^. LC and HCC outcomes after HBV infection were still not effectively controlled at this stage, such that continuous effective antiviral therapy and early recognition and diagnosis turned out to be the focus of clinical treatment.

Considerable risk assessment models have been adopted to predict LC and HCC in CHB patients (e.g., GAG–HCC score^32^, REACH–B score^33^, as well as the PAGE–B score^34^). GAG–HCC score predicts the occurrence of HCC in patients with hepatitis B within 5 and 10 years based on age, gender, albumin, bilirubin, HBV DNA and LC. REACH–B score predicts the occurrence of HCC in hepatitis B patients within 3, 5 and 10 years based on age, gender, HBeAg, HBV DNA and ALT. PAGE–B score predicts the occurrence of HCC in patients with hepatitis B within 5 years based on age, gender and platelet count. The above-mentioned risk scores tend to require patients to have complete and real medical record information, have some difficulties in practical clinical application, and predict less well than expected. ApoB/ApoA1 ratio refers to a common and easily obtainable indicator, showing a correlation with the prevalence of LC in CHB and the prevalence of HCC in LC. After various confounding factors were regulated, ApoB/ApoA1 ratio remained negatively correlated with the prevalence of both. In this study, ROC curves were generated. As indicated by the curves, combined diagnosis of ApoB/ApoA1 ratio and AFP exhibited the capability of improving the diagnostic efficacy and specificity of AFP for HCC, thus suggesting the clinical application value of combined diagnosis of ApoB/ApoA1 ratio and AFP. Studies have shown that not all hepatocellular carcinomas secrete high levels of AFP, and that high levels of AFP can be detected in the serum of some patients with cirrhosis and chronic hepatitis^35^, so in this paper, we set up an AFP-negative HCC group for differential diagnosis, and we found that the combined index of ApoB/ApoA1 ratio and AFP has a good diagnostic value. Thus, detection of ApoB/ApoA1 ratio takes on critical significance in identifying high-risk patients with HCC versus LC and effectively preventing CHB progression.

The role played by ApoB/ApoA1 ratio in the disease progression of CHB remains unclear, whereas multiple important mechanisms may be involved. Existing research has confirmed that HBV infection is capable of protecting infected subjects from the development of metabolic syndrome, and it shows a negative correlation with lipid metabolism^36^. The incidence of hypertriglyceridemia, hypercholesterolemia, and fatty liver in HBV infected individuals is relatively low. HBV infection increased fatty acid oxidation and reduced lipid droplets (LD) formation. The content of intracellular TGs and the mean size of individual LDs were significantly lower in HBV-infected or transfected cells compared to control cells due to reduced levels of proteins involved in LD expansion and lipid storage. HBV replication also increased adiponectin expression. Adiponectin alleviated lipid accumulation by increasing carnitine palmitoyl transferase 1 (CPT1) activity, enhancing hepatic fatty acid oxidation, and reducing ACC and FAS activity^20^.

Serum ApoB and ApoA1 decreased in CHB, HBV-associated LC, and HBV-associated HCC groups compared with healthy controls, while there were few comparative studies between the groups^21,37,38^. HBV can modulate ApoA1 function through HBx, leading to ApoA1 dysfunction (e.g., decreased self-association ability, elevated carbonyl levels, as well as impaired lipid binding ability^20,21^). Epigenetic silencing of ApoA1 gene expression by CpG island DNA hypermethylation induced by HBV can also reduce serum ApoA1 level^39^. HBV suppresses ApoB production by inhibiting microsomal triglyceride transfer protein expression^24^, overexpression of HBx leads to negative regulation of microsomal TG transfer proteins, which can increase the expression of β-d-mannoside-1, 4-N-acetylglucosaminyl transferase-III, and cause inhibition of ApoB secretion and intracellular accumulation of TG and TCh^40^.

The higher the ApoB/ApoA1 ratio, the more cholesterol will be deposited on the arterial wall, such that atherogenesis can be triggered, and cardiovascular disease risk will be elevated^16^. In terms of CHB patients, there are evidences of a relatively high risk of platelet activation and atherothrombosis^18,19^. Perhaps in the progression of CHB disease, HBV reduces the risk of atherosclerosis through the incompletely verified molecular mechanism, resulting in a negative correlation between ApoB/ApoA1 ratio and the prevalence of HCC and LC. It has been demonstrated that HBV-infected humanized mice show a significant increase in human genes correlated with cholesterol uptake, biosynthesis and transcriptional regulation, such as low-density lipoprotein receptor (LDLR), hydroxymethylglutaryl coenzyme A reductase (HMGCR), and HBV exacerbates hepatic cholesterol accumulation by up-regulating LDLR and HMGCR in HepG2 cells^41,42^. Sodium taurocholate cotransporting polypeptide (NTCP) is capable of mediating the passage of bile salts from the hepatic portal vein blood into hepatocytes. Besides, NTCP has been identified as a receptor mediating species-specific entry of HBV into hepatocytes. HBV infection plays a certain role in limiting NTCP function. Blocking bile acid uptake can facilitate compensatory up-regulation of cholesterol 7α-hydroxylase, such that cholesterol synthesis can be promoted in cells^43,44^. HBV infection promotes hepatic cholesterol accumulation and may lower the risk of atherosclerosis. Accordingly, with the progression of CHB disease, the risk of atherosclerosis can be lowered, and the ApoB/ApoA1 ratio can be down-regulated. ApoB/ApoA1 ratio may serve as a potential marker to lay a novel basis for early identification of LC and HCC in CHB patients.

### Study strength and limitations

This study first aimed to verifying that the ApoB/ApoA1 ratio is strongly correlated with CHB disease progression, and its predictive potential is not correlated with potential risk factors (e.g., age, anti-hepatitis treatment, and duration of chronic hepatitis). Thus, the above-mentioned findings have high clinical utility for the identification of high-risk patients with HCC or LC.

However, several limitations remained in this study. First, all serum samples were from Chinese, suggesting that the generalizability of the findings in different regions or ethnic groups should be validated in depth. Second, the statistical power of this study was limited by the small sample size and may be biased. Third, prognostic and survival studies were hindered by the lack of long-term follow-up data in this study. Fourth, the confounding factors regulated for in this study may not be comprehensive.

## Conclusion

In brief, low serum ApoB/ApoA1 ratio was correlated with an elevated risk of LC and HCC in CHB patients. Notable difference was reported in serum ApoB/ApoA1 ratio between LC and HCC patients at different stages, and combined diagnosis with AFP exhibited the capability of increasing the detection efficacy and specificity of AFP for HCC and AFP-negative HCC. As revealed by the above-mentioned findings, the serum ApoB/ApoA1 ratio in CHB patients may be conducive to identifying high-risk patients for HCC or LC, such that LC and HCC can be early diagnosed and treated.

## Data Availability

The datasets generated during and analyzed during the current study are not publicly available due to privacy or ethical restrictions but are available from the corresponding author on reasonable request.

## Abbreviations

LC: Liver cirrhosis
HCC: Hepatocellular carcinoma
CHB: Chronic hepatitis B
TG: Triglyceride
TCh: Total cholesterol
LDL-C: Low-density lipoprotein cholesterol
HDL-C: High-density lipoprotein cholesterol
SdLDL-C: Dense low-density lipoproteinsd cholesterol
ApoB: Apolipoprotein B
ApoA: Apolipoprotein A1
ALT: Alanine aminotransferase
AST: Aspartate aminotransferase
ALP: Alkaline phosphatase
GGT: Alanine aminotransferase
Fn: Fibronectin
CHE: Cholinesterase
FFA: Free fatty acid
LDH: Lactate dehydrogenase
PEG: Polyethylene glycol
AFP: Alpha fetoprotein
MELD: Model for end-stage liver disease
LP(a): Lipoprotein a
OR: Odds ratio
HMGCR: Hydroxymethylglutaryl coenzyme A reductase
LDLR: Low-density lipoprotein receptor
NTCP: Sodium taurocholate cotransporting polypeptide
